# Diurnal suppression of EGFR signalling by glucocorticoids and implications for tumour progression and treatment

**DOI:** 10.1038/ncomms6073

**Published:** 2014-10-03

**Authors:** Mattia Lauriola, Yehoshua Enuka, Amit Zeisel, Gabriele D’Uva, Lee Roth, Michal Sharon-Sevilla, Moshit Lindzen, Kirti Sharma, Nava Nevo, Morris Feldman, Silvia Carvalho, Hadas Cohen-Dvashi, Merav Kedmi, Nir Ben-Chetrit, Alon Chen, Rossella Solmi, Stefan Wiemann, Fernando Schmitt, Eytan Domany, Yosef Yarden

**Affiliations:** 1 Department of Biological Regulation, Weizmann Institute of Science, Rehovot 76100, Israel; 2 Unit of Histology, Embryology and Applied Biology, Department of Experimental, Diagnostic and Specialty Medicine, Bologna University, Bologna 40138, Italy; 3 Department of Physics of Complex Systems, Weizmann Institute of Science, Rehovot 76100, Israel; 4 Division of Molecular Genome Analysis, German Cancer Research Centre (DKFZ), 69120 Heidelberg, Germany; 5 Department of Neurobiology, Weizmann Institute of Science, Rehovot 76100, Israel; 6 Department of Laboratory Medicine and Pathobiology, Faculty of Medicine, University of Toronto, Toronto, Ontario, Canada M5S 1A8; 7 Department of Pathology, University Health Network, Toronto, Ontario, Canada M5G 2C4; 8 IPATIMUP, University of Porto, Porto 4200-465, Portugal

## Abstract

Signal transduction by receptor tyrosine kinases (RTKs) and nuclear receptors for steroid hormones is essential for body homeostasis, but the cross-talk between these receptor families is poorly understood. We observed that glucocorticoids inhibit signalling downstream of EGFR, an RTK. The underlying mechanism entails suppression of EGFR’s positive feedback loops and simultaneous triggering of negative feedback loops that normally restrain EGFR. Our studies in mice reveal that the regulation of EGFR’s feedback loops by glucocorticoids translates to circadian control of EGFR signalling: EGFR signals are suppressed by high glucocorticoids during the active phase (night-time in rodents), while EGFR signals are enhanced during the resting phase. Consistent with this pattern, treatment of animals bearing EGFR-driven tumours with a specific kinase inhibitor was more effective if administered during the resting phase of the day, when glucocorticoids are low. These findings support a circadian clock-based paradigm in cancer therapy.

## Abstract (web summary)

Glucocorticoids are released in a diurnal pattern. Here, the authors show that EGF receptor (EGFR) signalling is negatively regulated by glucocorticoids, and that EGFR inhibitor has stronger antitumour effects when administered during the resting phase, when glucocorticoids are low, offering potential optimization of cancer therapy regimens.

## Graphical abstract

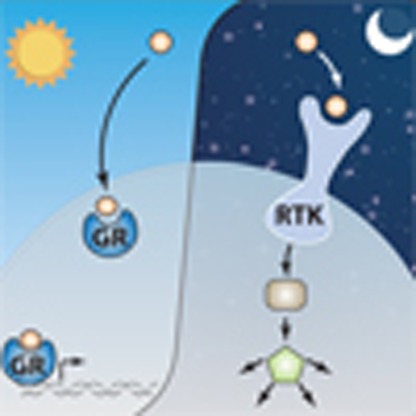

Growth factors acting through receptor tyrosine kinases (RTKs), along with steroid hormones acting through nuclear receptors (NRs), critically regulate cell-to-cell interactions in development and throughout adulthood. For example, type I RTKs (also called ERBB or HER) and their ligands of the epidermal growth factor (EGF) family regulate ductal and alveolar morphogenesis of the mammary gland^1^. Similarly, the NR called glucocorticoid (GC) receptor (GR) controls cell proliferation during lobulo-alveolar development of the mammary gland^2^. Despite recruitment of very different routes of signal transduction, RTKs and NRs maintain extensive cross-talk. For example, the oestrogen receptor (ER) acts as a transcription factor (TF) that mediates the response to oestrogens and to some anti-cancer therapies, including Tamoxifen^3^. ER is modulated by several RTKs, such as EGFR, HER2 and the insulin-like growth factor receptor^4^. The overexpression or stimulation of these RTKs can activate the downstream mitogen-activated protein kinase (MAPK)/ERK and phosphoinositide3-kinase (PI3K)/AKT pathways, which activates specific transcriptional programmes. The activation of these downstream pathways has been associated with phosphorylation of ER at multiple serine residues^5^.

One prototypical RTK is the EGF receptor (EGFR/ERBB1). In addition to EGF, EGFR binds several growth factors, including transforming growth factor-α (TGFA) and the heparin-binding EGF-like growth factor (HB-EGF)^6^. Integration of EGF-induced signals culminates in a wave-like pattern of transcription^7^: in response to EGF, a group of microRNAs undergoes rapid downregulation, and concurrently their target transcripts, which encode immediate early TFs (IETFs), and other immediate early genes are activated. Subsequent transcription of delayed early genes, a group encoding transcriptional repressors and negative feedback regulators, such as MAPK phosphatases (DUSPs) and ERRFI1/MIG6, which promotes degradation and inhibits self-phosphorylation of EGFR^8^, regulates expression of late, fate-determining genes.

In analogy to RTKs, the biological actions of GCs, as well as other steroid hormones, are mediated by ubiquitously expressed receptors of the NR superfamily^9^. GCs are synthesized in the adrenal gland and are delivered through systemic circulation to GRs^10^. Once in the nucleus, ligand-bound GRs activate transcription by binding to specific DNA elements, called GC response elements (GREs). Alternatively, GR mediates direct repression of specific genes by binding to negative GREs (nGREs)^11^ or by altering chromatin state^12^. Yet, an additional mechanism of regulation involves tethered transrepression by physical complex formation between GRs and other TFs, such as signal transducer and activator of transcription 5 (STAT5; ref. 13). These modes of regulation mediate both prosurvival effects on epithelial cells and induction of apoptosis of lymphoid and myeloid cells, which led to the approval of a GC analogue some 50 years ago, for treatment of childhood leukaemia^14^. GCs are also widely used as co-medication of various carcinomas, due to their ability to reduce toxicity of chemotherapy^15^. Additional therapeutic applications may arise from better understanding of the roles for GCs in daily rhythms. Adrenal secretion of GCs fluctuates in a circadian and stress-related manner and disruption of circadian rhythm was found to accelerate tumour growth in animals^16^. Likewise, stressful conditions were associated with more aggressive mammary tumorigenesis in rats^17^, but these relationships remain unclear in humans. Thus, better understanding of circadian controlled factors, such as steroids, melatonin and growth factors, holds promise for cancer treatment^18^.

The present study was motivated by our observation that GR can inhibit EGF-induced mammary cell migration. The underlying mechanism involves repression of well-known activators of EGFR signalling, alongside with enhancement of several EGFR’s negative feedback loops. In particular, we found that the EGFR—GR cross-talk entails reciprocal regulation of the MAPK pathway. Our animal studies confirmed daily antithetical oscillation of EGFR’s feedback loops, and analyses of clinical specimens uncovered association between high GR, low MAPK activity and favourable prognosis of breast cancer patients. Moreover, administration of an anti-EGFR drug during the active phase of the day, rather than the resting time, less effectively inhibited tumour growth in animals. These findings and the emerging daily GR-to-RTK cross-talk call for circadian rhythm-based scheduling of anticancer drugs.

## Results

### Ligand-activated GRs inhibit EGF-induced cell motility

On stimulation with EGF, MCF10A mammary epithelial cells initiate transcriptional programmes culminating in migration and invasion^19^,^20^. To examine potential interactions between the EGFR pathway and steroid hormone signalling, we plated cells in transwell trays and treated them with EGF, in the presence of estradiol (E2), progesterone (PRG), medroxyprogesterone acetate (MPA), a synthetic variant of PRG, or dexamethasone (DEX), a synthetic GC (Supplementary Fig. 1A). The results identified DEX as a potent inhibitor of EGF-induced cell migration, and experiments that are not presented confirmed that hydrocortisone (HC) acted similarly. MPA was less potent, and both E2 and PRG displayed weak or no activity, probably due to the absence of the respective receptors. Importantly, markers of apoptosis (annexin V) and necrosis (propidium iodide) excluded the possibility of cellular toxicity (Supplementary Fig. 1B).

**Figure 1:**
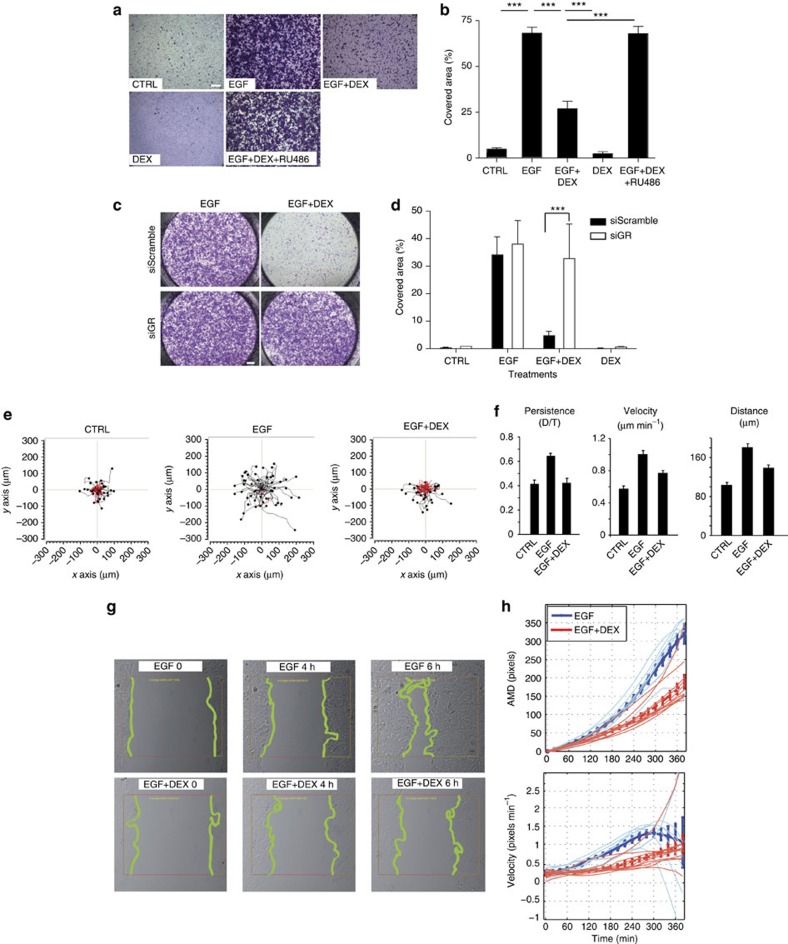
Ligand-bound GRs inhibit inducible migration of mammary cells. (**a**) MCF10A cells growing in transwells were treated for 16 h with EGF (10 ng ml^−1^), DEX (100 nM), RU486 (5 μM) or their combinations. Shown are representative crystal violet staining images of migrated cells from three experiments. Scale bar, 500 μm. (**b**) Cell-covered areas from four microscope fields of **a** were determined. ***P<0.0001 (one-way analysis of variance (ANOVA)). (**c**) Cells pretreated with the indicated siRNA oligonucleotides were seeded in transwells, stimulated as shown and 16 h later migrated cells were photographed. (**d**) Quantification of results from **c**. ***P<0.001 (one-way ANOVA). (**e**) MCF10A cells treated with EGF or DEX were followed using time-lapse microscopy. Shown are rose plots of single-cell trajectories; red tracks indicate migration persistence smaller than 0.3. (**f**) Quantification of migration parameters from **e** (means±s.e.m.; from 60 cells). (**g**) Wound closure assays were performed following the indicated treatments of MCF10A cells. Green lines mark migration fronts. This experiment was repeated twice. (**h**) Quantification of time-lapse movies from **g**. Five-minute frames were used (fine lines) and both average migration distance and velocity are presented.

Although GCs interact with both GR and the mineralocorticoid receptor (MR), MCF10A cells, similar to the majority of mammary cell lines^21^, express no detectable MR. In addition, MPA binds the PRG, androgen and GRs with EC50 values of ~0.01, 1 and 10 nM, respectively. Hence, migration inhibition by DEX and, to some extent, by MPA could be mediated by GR, as supported by using RU486, a GR antagonist (Fig. 1a,b). In addition, GR-specific small interfering RNAs (siRNAs) that reduced receptor expression by ~80% (Supplementary Fig. 1C) inhibited the effect of DEX on migration (Fig. 1c,d). These results raised the possibility that ligand-bound GRs inhibit EGF-induced cell migration by modulating transcriptional events. Yet, as DEX might activate MRs, which are PCR undetectable, we cannot exclude involvement of MRs in the antagonism of EGFR-mediated effects on MCF10A cell migration.

Next, we asked whether DEX treatment alters migration directionality, namely the ability of cells to maintain a migration course^22^. Quantification of directionality relates the linear distance between the start and end points (D) to the total distance (T) travelled. The rose plots of cellular tracks (Fig. 1e,f) indicated that EGF enhanced directional persistence (D/T) and accelerated velocity, but DEX abolished these effects. To complement these observations, we performed a wound closure assay, as recently described^23^ (Fig. 1g,h). The results strengthened the possibility that GR activation interferes with EGF-induced migration.

### Comparison of gene programmes stimulated by GR and EGFR

**Figure 2:**
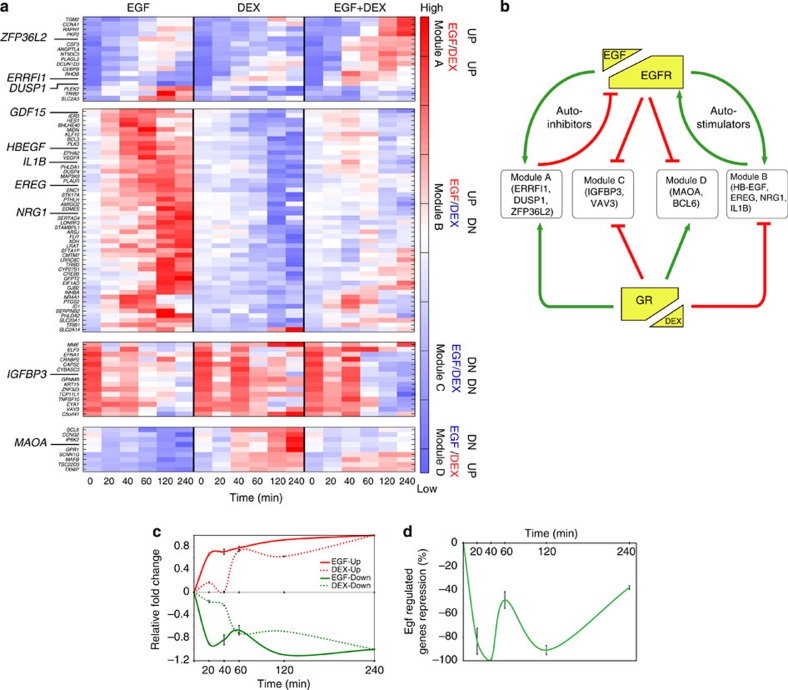
Activated GRs repress EGF-induced transcriptional programmes. (**a**) RNA was isolated from MCF10A cells that were pretreated as indicated and hybridized to Affymetrix Exon Arrays. The heatmaps display RNA fold changes, which were clustered into four groups and ordered according to RNA’s peak time. (**b**) A scheme depicting relationships among EGFR, GR and the four gene modules. (**c**) For each time point, we calculated the average fold changes in gene expression (EGF only versus DEX only) relative to *t*=240 min. The red lines indicate upregulated genes, whereas the green lines show the downregulated genes. (**d**) The average difference between the fold change following EGF treatment and the ‘DEX plus EGF’ treatment was used to present the extent of repression relative to *t*=40 min.

Conceivably, the inhibitory effect of GR involves alterations of EGF-induced transcription. Specifically, GR might affect transcript synthesis or modulate EGF-induced RNA splicing in MCF10A cells. To address such models, we stimulated cells with EGF, DEX or the combination, isolated messenger RNAs along a time course from 20 min to 4 h, and hybridized the RNA to Affymetrix Exon Arrays able to resolve small changes in splicing^24^. The results we obtained are summarized in Fig. 2a. In addition, Supplementary Fig. 2 presents confirmatory PCR analyses. Notably, the combined treatment exerted no marked effects on RNA splicing. To cluster other transcriptional events, we applied a set of logical rules and defined modules of active genes (Fig. 2b):

Module A: Transcripts upregulated by both EGF and DEX (EGF^UP^/DEX^UP^)

Module B: Transcripts upregulated by EGF but downregulated by DEX (EGF^UP^/DEX^DN^)

Module C: Transcripts downregulated by both agents (EGF^DN^/DEX^DN^)

Module D: Transcripts downregulated by EGF and upregulated by DEX (EGF^DN^/DEX^UP^).

Interestingly, we noted that Module A (EGF^UP^/DEX^UP^) included several inducible inhibitors of EGFR, such as *ERRFI1/MIG6*, *ZFP36L2* and *DUSP1*, which are normally engaged in delayed feedback inhibition of EGFR signalling^8^. Conceivably, their induction by GR represents an effective inhibitory strategy. Consistent with this logic, Module B (EGF^UP^/DEX^DN^) includes positive feedback regulators of the EGFR pathway, such as neuregulin 1, HB-EGF and EREG, which sustain EGFR signalling^25^. In conclusion, GR orchestrates a transcriptional response resulting in downregulation of several positive EGFR regulators (Module B) coupled with upregulation of multiple EGFR inhibitors (Module A), thereby robustly terminating EGFR signalling.

Comparison of the temporal patterns of EGF- and DEX-regulated genes indicated that the onset of EGF-induced or repressed transcripts was very fast in comparison with the effect of DEX (when applied alone). The major effect of the latter stimulant displayed a 40-min long delay and peaked at 60 min (Fig. 2c). Next, we addressed the effect of a mixture of EGF and DEX on EGF-induced transcripts (upregulated) (Fig. 2d). This analysis revealed that the maximal suppressive effect of DEX has an impact on the genes that undergo early (<40 min) induction by EGF: the inhibitory effect of DEX reached 70% of maximal suppressive capacity already at 20 min (Fig. 2d), significantly earlier than the peak of changes induced by DEX alone. Altogether, these observations raised the possibility that GR intercepts specific TFs that undergo post-translational modifications downstream to EGFR signalling.

### GR exploits a feedback module that terminates RTK signalling

**Figure 3:**
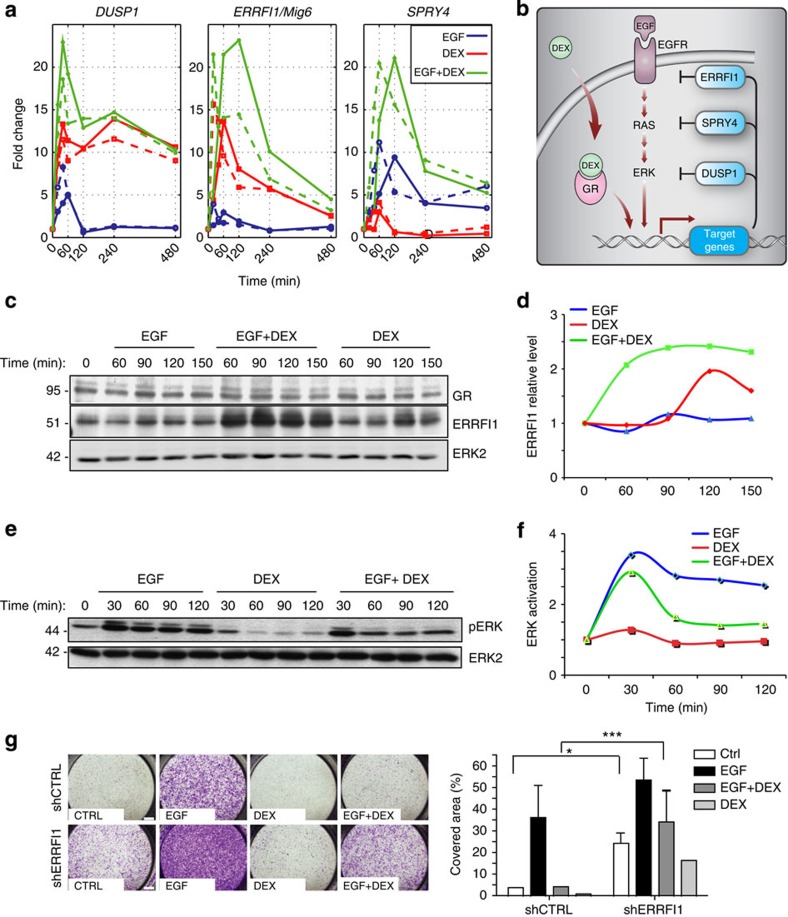
GR induces negative feedback regulators of EGFR signalling. (**a**) Serum-starved MCF10A cells were treated with EGF or DEX. Quantitative PCR analysis was performed using RNA and primers corresponding to pre-mRNAs (dashed lines) or the mature forms (solid lines). (**b**) A scheme depicting negative feedback regulators of EGFR signalling. (**c**,**d**) Cells were stimulated as in **a** and extracts were immunoblotted for ERRFI1, GR and ERK2. Normalized ERRFI1 signals are shown. (**e**,**f**) Active ERK signals (phosphorylated ERK (pERK)) were determined, normalized and presented. Images have been cropped for presentation; full-size images are presented in Supplementary Fig. 7. (**g**) MCF10A derivatives stably expressing ERRFI1-specific short hairpin RNAs (shRNAs) were tested for migration following the indicated treatments. Quantification of the results used one-way analysis of variance. *P<0.05; ***P<0.0001.

Analyses of defects in vulva formation in worms and aberrations in eye development in insects, two processes controlled by EGFR, helped define several evolutionary conserved and partly redundant negative feedback loops able to robustly terminate EGFR signalling^26^. As Module A (EGF^UP^/DEX^UP^) includes several orthologues of the invertebrate negative feedback loops, we selected three for further analysis. DUSP1 is the prototype of MAPK-specific phosphatases, which dephosphorylate the shared Thr-Xxx-Tyr motif of MAPKs. ERRFI1/MIG6 (also called RALT) is a previously identified steroid-inducible adaptor, which physically binds and inhibits the kinase domain of EGFR^8^. The third feedback regulator we studied was sprouty 4, a member of the small family of adaptors able to specifically inhibit RAS-to-ERK signalling^27^. By using primers specific for the nascent or the mature transcripts, we followed *de novo* transcription of these three negative regulators (Fig. 3a). The precursor and mature transcripts exhibited similar profiles, but unlike the relatively transient and weak induction of *DUSP1* and *ERRFI*1 by EGF (3- to 5-fold), treatment of cells with DEX, and especially with the DEX+EGF combination, strongly enhanced and prolonged the upregulation signal (20- to 25-fold). As each feedback regulator acts at a different level of the signalling cascade (Fig. 3b), and all three were rapidly induced, the enhanced and prolonged induction by the DEX+EGF combination probably translates to robust inhibition of the RTK-to-ERK signalling pathway. This possibility was further examined by focusing on *ERRFI1*.

Consistent with the gene expression data, immunoblotting confirmed strong upregulation of the ERRFI1 protein in cells co-treated with EGF and DEX (Fig. 3c). Similarly, quantification of the signals indicated that the combined treatment induced an earlier and more sustained activation of ERRFI1 (Fig. 3d). As the three regulators we selected for analysis act upstream to ERK, we examined the status of active ERK (phosphorylated ERK; Fig. 3e,f). EGF rapidly stimulated ERK, but the addition of DEX reduced the amplitude and markedly shortened the duration of ERK activation. This effect appeared to depend on *de novo* transcription, as DEX was unable to reduce ERK activation in the presence of a transcription inhibitor, actinomycin D (Supplementary Fig. 3).

To monitor functional consequences of the GR-to-RTK cross-talk, we stably reduced ERRFI1 expression and tested the ability of DEX to inhibit EGF-induced migration (Fig. 3g). Interestingly, under basal (unstimulated) conditions *ERRFI1*-depleted cells displayed higher migration relative to the control cells. Nevertheless, EGF still increased migration of *ERRFI1*-depleted cells, but the inhibitory effect of DEX was much smaller compared with control cells. Whereas DEX inhibited migration of control cells by 90%, this effect was diminished to 30% in *ERRFI1*-depleted cells. In conclusion, GR activation involves upregulation of a well-characterized group of negative feedback regulators of EGFR signalling. In line with the critical roles played by EGFR’s feedback regulators in GR signalling, intercepting the function of just one of these regulators, *ERRFI1*, significantly limited the ability of GR to inhibit EGFR signalling.

### GR transcriptionally represses EGFR’s positive feedback

**Figure 4:**
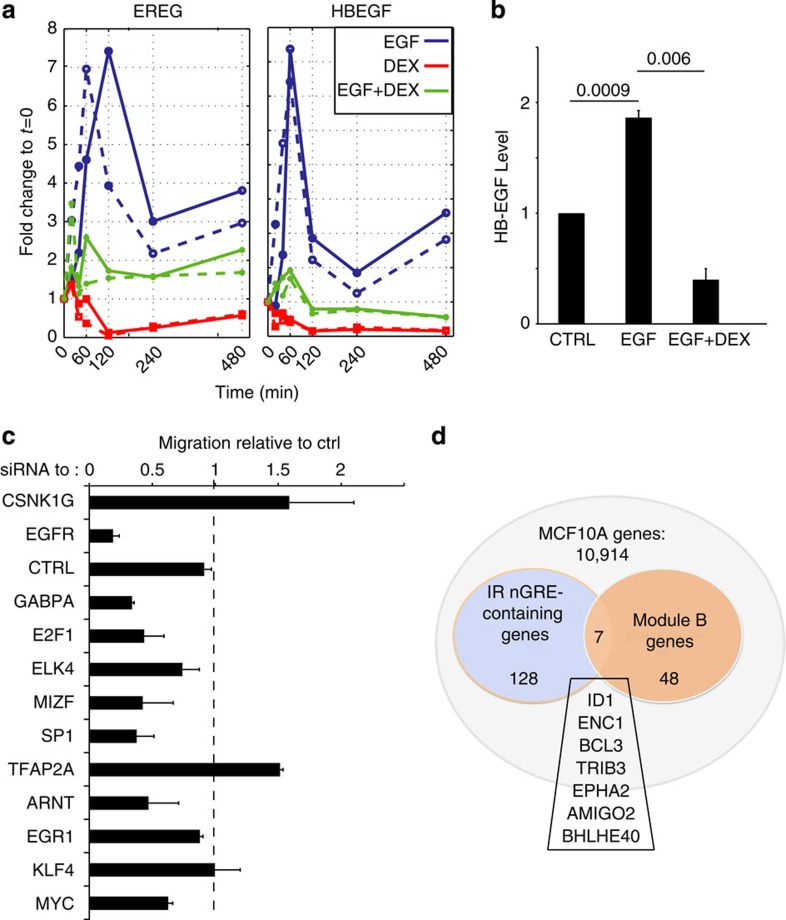
GR rewires EGFR programmes via IR nGREs and transrepression. (**a**) MCF10A cells were analysed for expression of the indicated genes as in Fig. 3a. (**b**) Cells were treated for 4 h, as indicated, and extracts were tested for HB-EGF using enzyme-linked immunosorbent assay. Results represent biological duplicates performed in technical triplicates. *P*-values (indicated above the horizontal lines) were calculated using one-way analysis of variance and Tukey’s multiple comparison test. (**c**) The indicated siRNAs were transfected into MCF10A cells, which were re-seeded 48 h later, scratched and stimulated with EGF. Migration (average±s.e.m.) was assayed 12 h later in triplicates. (**d**) Distribution of genes expressed by MCF10A cells, including IR nGRE-containing, DEX-downregulated genes and Module B genes. The overlapping seven genes were obtained by using a hypergeometric test (*P*=1.28 × 10^*−*6^).

EGF-dependent transcriptional responses are characterized by early induction of auto-stimulatory loops comprising several growth factors, such as *TGFA*, *NRG1*, *EREG* and *HBEGF*, which not only auto-stimulate EGFR but also engage additional EGFR family members^25^. DEX strongly inhibited these auto-stimulatory loops, as detected by real-time and immunological assays (Fig. 4a,b). The observed rapid effects of DEX on the levels of both pre-mRNA and mRNA levels raised the possibility that GR transrepresses pre-existing IETFs responsible for regulation of EGFR ligands and other module B genes. To examine this, we first searched for TF binding motifs overrepresented in the promoters of Module B genes, and then validated the results by using Cscan^28^, a software based on extensive chromatin immunoprecipitation experiments (Table 1). In the next step, we functionally tested each protein of the resulting list by using siRNAs and a migration assay. The results presented in Fig. 4c indicated that depletion of the majority of candidates reduced EGF-induced migration, in line with a transrepression model that repeatedly engages a relatively small group of TFs to inhibit EGFR signalling. Interestingly, some of the predicted TFs, such as GABPA, ELK1 and ELK4, belong to the ETS family, which is directly regulated by the MAPK pathway, while others (for example, SP1 and E2F1) are differently regulated by growth factors.

Along with physical tethering of specific TFs, such as nuclear factor-κB (NF-κB) and STAT5 (ref. 13), GR might induce direct repression via binding to palindromic sequences consisting of two inverted repeated motifs, inverted repeated (IR) nGREs, which are *cis*-acting response elements^11^. Probing MCF10A cells, we identified 128 genes that contain IR nGRE in their regulatory regions (~1% of all expressed genes). Astonishingly, by focusing only on the Module B genes, the enrichment for IR nGREs reached 15% (*P*=1.2781e−06; Fig. 4d). For example, this group encodes BCL3, which regulates NF-κB target genes^29^. In conclusion, these findings offer two GR-mediated modes of suppressing RTK signalling: first, by transrepressing pre-existing TFs, and second by binding to IR nGREs.

### Daily GC oscillations control EGFR’s gene programmes *in vivo*

**Figure 5:**
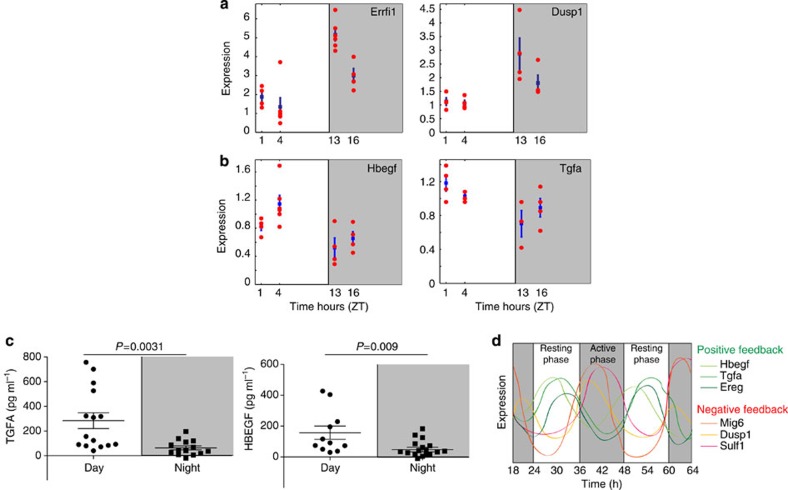
Diurnal control of EGFR transcriptional programmes in animals. (**a**,**b**) Mouse livers (*n*=4) were collected at the indicated time of the day or night (grey areas) and analysed using reverse transcriptase–PCR for *ERRFI1* and *DUSP1* (negative regulators) or *HBEGF* and *TGFA* (positive regulators). Zeitgeber (ZT) zero indicates light ON. (**c**) Serum from WT mice was collected at ZT4 and ZT10 (‘day’), or ZT15 and ZT20 (‘night’), and assayed using enzyme-linked immunosorbent assay for TGFA and HBEGF. (**d**) Composite panel of experimentally determined antithetical oscillations of EGFR’s negative (Mig6, Dusp1 and Sulf1) and positive feedback regulators (Tgfa, Hbegf and Ereg) as reported in the Circa DB gene expression database (http://bioinf.itmat.upenn.edu/circa/query). The following murine tissues were used as sources of RNA during the active and resting phases: the liver, pituitary, brain stem and brown adipose.

Next, we explored *in vivo* the cross-talk between GR and the RTK pathway. GCs exhibit a daily rhythm, which affects behavioural patterns^30^, and this oscillation has generally been attributed to the hypothalamus–pituitary–adrenal (HPA) neuroendocrine axis. The oscillation profile has a characteristic pattern, with a peak in the beginning of the active, dark phase in rodents. To examine the prediction that GCs control expression of EGFR’s negative regulators, we analysed mRNA levels of two Module A genes, *Errfi1* and *Dusp1*, in mouse organs. As analyses of diurnal regulation of gene expression often employ liver as the test organ, and due to the abundance of hepatic tissue available for RNA sampling, our assays focused on mouse livers. It is noteworthy that we also tested lungs and intestinal tissues, and obtained results similar to those obtained with livers. In support of a suppressive cross-talk, *Errfi1* and *Dusp1* displayed daily oscillations with amplitudes of two- to fourfold change and higher levels in the active, nocturnal phase (Fig. 5a). By contrast, two EGFR-positive regulators, *Hbegf* and *Tgfa*^31^, displayed reciprocal patterns, peaking during the resting (diurnal) phase (Fig. 5b). Using enzyme-linked immunosorbent assay and mouse blood samples collected during the diurnal (ZT4–ZT10) and nocturnal (ZT15–ZT20) phases, we supported the possibility that the levels of HB-EGF and TGFA oscillate in a circadian manner (Fig. 5c). Furthermore, compilation of experimental data from expression arrays available through Circa DB, the circadian expression profiles database (http://bioinf.itmat.upenn.edu/circa/query), confirmed antithetical oscillations of EGFR’s negative (*Mig6*, *Dusp1* and *Sulf1*) and positive (Tgfa, Hbegf, Ereg) feedback regulators, as determined by analysing a set of four different murine tissues (Fig. 5d). In summary, both positive and negative feedback regulators of RTK signalling display oscillatory patterns *in vivo*, in line with diurnal secretion of the activators, namely EGFR ligands, coupled to nocturnal synthesis of several intracellular inhibitors of EGFR signalling, to achieve robust suppression and activation of EGFR signalling during the active (nocturnal) and resting (diurnal) phases, respectively, in rodents.

**Figure 6:**
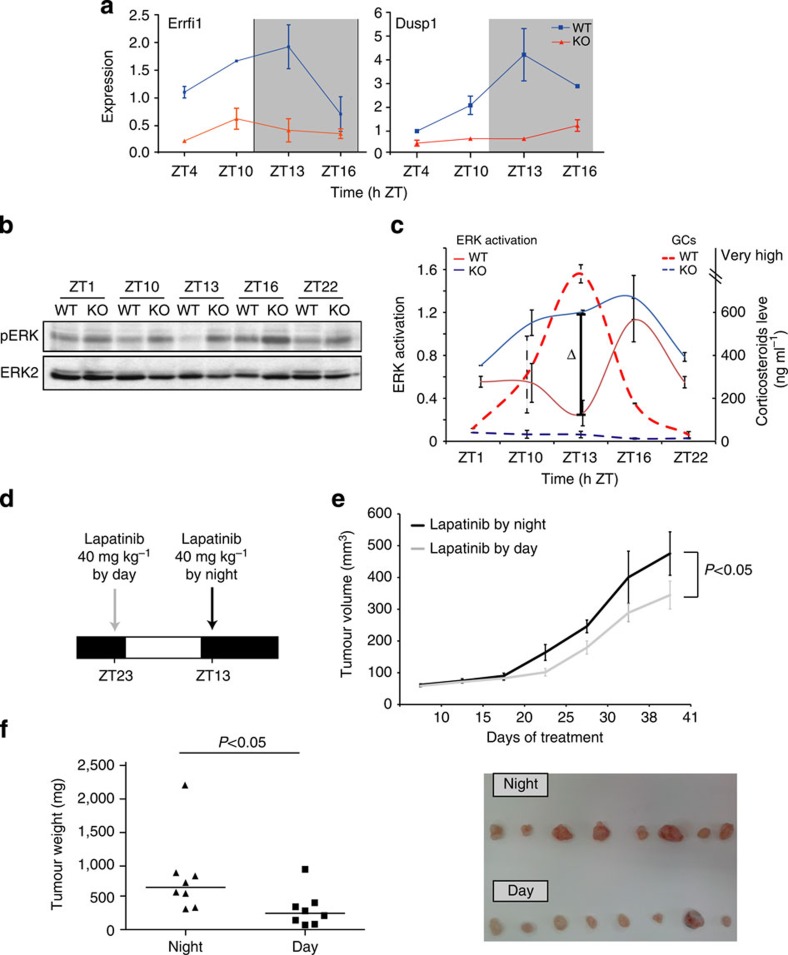
Circadian oscillations control EGFR signals and tumour growth. (**a**) WT and CRFR1^*−*/*−*^ (knockout (KO)) mice were killed at the indicated times and liver mRNA was extracted. Errf1 and Dusp1 were assayed using reverse transcriptase–PCR. (**b**) The status of ERK activation in WT and CRFR1^*−*/*−*^ (KO) mice was determined using immunoblotting of liver extracts. (**c**) The normalized level of ERK activity (from **b**) is plotted, along with the corresponding corticosteroid serum concentration, as detected by using a radioimmunoassay (dashed lines; very high refers to saturation of the assay). Δ The lowest point of ERK activity corresponding to the peak of GCs in WT mice. (**d**–**f**) CD1/nude mice were injected subcutaneously with 5 × 10^6^ N87 cells. Lapatinib treatment (40 mg kg^−1^ per day) was started once tumours became palpable, about 2 weeks after the inoculation. The ‘day’ group received Lapatinib by oral gavage just before the beginning of the resting phase, while the night group received oral gavage Lapatinib at the beginning of the active phase (see a scheme). Tumour sizes±s.e.m. are presented. In the end of the experiment, tumours were weighted (each dot represents one animal) and photographed.

To corroborate these conclusions, we looked for a murine model with aberrant GC production. CRFR1 encodes one of two receptors for the corticotrophin releasing factor, which maintains the HPA axis^32^. Homozygous Crfr1-depleted mice (*Crfr1*^*−/−*^) display constantly low plasma corticosterone concentrations resulting from agenesis of the zona fasciculata region of the adrenal gland^33^. Hence, this animal model represents a suitable system for addressing the GR-to-RTK cross-talk. Consistent with other lines of evidence, the expression levels of two negative feedback regulators, *Errfi1* and *Dusp1*, were generally reduced in the livers isolated from *Crfr1*-deficient mice and they lacked the circadian fluctuations observed in control mice (Fig. 6a). These results suggested that EGFR signalling is under control of the HPA neuroendocrine axis. However, as CRFR’s ligands have effects besides those on the HPA axis, we performed the following rescue experiments: For 7 days, we supplied HC to Crfr1^*−*/*−*^ mice in the drinking water, and subsequently we assayed the *in vivo* patterns of two murine genes: a negative feedback regulator of EGFR (*Mig6/Errfi1*) and a positive feedback component (*Hbegf*). The results we obtained using a published protocol^34^ indicated that HC supplementation rectified the phenotype of Crfr^*−*/*−*^ mice (Supplementary Fig. 4); although *Hbegf* was downregulated in HC-treated knockout mice (relative to untreated Crfr1^*−*/*−*^ animals), *Mig6* was upregulated, in line with suitability of the animal model.

In the next step, we tested ERK activation, a downstream effector of EGFR, in liver extracts collected around the clock from wild-type (WT) and mutant animals (Fig. 6b,c). Interestingly, the *Crfr1* mutant mice displayed normal ERK activation, but they lacked the inactivation phase (marked by Δ in Fig. 6c), which coincides with the peak of corticosteroid concentration in blood. Moreover, in line with the suppressive action of GR, ERK displayed overall higher levels in the mutants compared WT animals. Altogether, the comparison between WT and *Crfr1* mutant mice supported the possibility that negative modulators of EGFR (that is, *Errfi1*) and MAPK (that is, *Dusp1*) are controlled *in vivo* by ligands of GR.

### Better responses to an EGFR drug administered during daytime

Constitutive signals generated by EGFR and its kin, called HER2 or ERBB2, drive several types of tumours, and drugs intercepting these signals are active in patients whose tumours display aberrant forms of these RTKs^35^,^36^. Lapatinib, an oral low-molecular-weight drug approved for breast cancer treatment, specifically inhibits the tyrosine kinase activities of both EGFR and HER2 (ref. 37). Our working hypothesis predicted that administration of Lapatinib at the beginning of the resting (diurnal) phase to mice carrying EGFR/HER2-driven tumours would better inhibit tumorigenic growth relative to administration during the active phase, in which EGFR signalling is anyhow robustly suppressed by liganded GRs. Notably, MCF10A cells are not tumorigenic and many tumorigenic mammary cells require oestrogen for their survival. Hence, we selected for *in vivo* analyses an alternative cell model, namely N87 human gastric cancer cells, which are sensitive to EGFR/HER2-targeting drugs^38^. Notably, employing different cells *in vivo* (gastric) and *in vitro* (mammary) is a disadvantage dictated by the lack of a suitable breast cancer cell line. Importantly, we confirmed that N87 cells responded to EGF and DEX in a way that resembled the responses of MCF10A cells in terms of two feedback regulators (Supplementary Fig. 5). Likewise, we confirmed that the gastric cells, such as mammary cells, enhanced their migration in response to EGF, but treatment with DEX inhibited their migration. In the next step, mice (CD1/nude) were injected subcutaneously with N87 cells, and once tumours became palpable we randomized the animals into several groups. The ‘day’ group received Lapatinib, just before the beginning of the resting phase, while the ‘night’ group was treated at the beginning of the active phase (see a scheme in Fig. 6d), both by oral gavage. Tumour volumes and body weights were followed over a period of several weeks, and tumour weights were inspected in the end of the trial (Fig. 6e,f). The results confirmed statistically significant enhancement of Lapatinib’s therapeutic impact when administered just before the resting phase (ZT23), and excluded adverse effects on body weight.

Interestingly, tumours differed not only by their size but also by their appearance, suggesting that the administration of Lapatinib during the resting phase also inhibited tumour angiogenesis (Fig. 6f, right panel), in line with a similar effect of an anti-HER2 antibody when tested in animals^39^. Taken together with the *in vitro* studies and observations made with genetically modified mice, the effect of timing on drug efficacy not only adds another line of evidence in support of our model, but also proposes a potential strategy capable of augmenting the therapeutic effects of anticancer drugs.

### High GR associates with good prognosis of breast cancer

**Figure 7:**
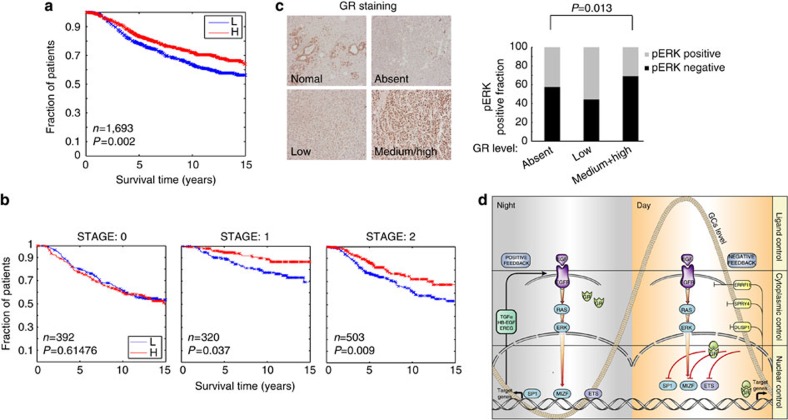
High GR associates with better prognosis of breast cancer. (**a**) Breast cancer specimens from the METABRIC data set were classified into two equal size groups according to GR transcript levels (high and low). The respective relapse-free survival (RFS) of each group is shown. (**b**) Breast cancer patients were divided into three groups according to tumour stage, and patient survival was analysed relative to GR abundance. (**c**) Shown are representative sections of GR immunostaining of invasive breast carcinomas (331 patients). The fraction of phosphorylated ERK (pERK)-positive specimens in each group was determined (*P*=0.013; *χ*^2^-test). (**d**) A model depicting the cross-talk between EGFR and GR during the active phase (right; high GC level) and the resting phase (left; low GC). Both positive and negative feedback loops regulating EGFR signalling are indicated, and signalling is divided into three layers.

As EGFR and other RTKs play pivotal roles in progression of human breast cancer^6^, and as our results indicated that GR signalling suppresses RTKs, we addressed GR’s prognostic significance in tumour specimens. To this end, we used a breast cancer data set that represents a collection of over 2,000 clinically annotated primary breast cancer specimens from tumour banks in the United Kingdom and Canada (the METABRIC data set^40^). Nearly all ER-positive and/or lymph node-negative patients did not receive chemotherapy, whereas ER-negative and lymph node-positive patients did. In addition, none of the HER2-positive patients received Trastuzumab. As such, the treatments were homogeneous with respect to clinically relevant groupings. Analysis of these clinical data associated high abundance of GR (*NR3C1*) with longer patient survival time (*P*=0.002; Fig. 7a). These relations were confirmed in two independent, but smaller groups of patients (Supplementary Fig. 6A). Notably, a previous study associated longer relapse-free survival with higher GR expression in a group of 87 patients, but this was limited to ER-positive patients^41^. Interestingly, stratifying patients of the METABRIC cohort according to disease stage indicated that low GR expression might predict poor survival only in advanced stages of disease (Fig. 7b), and similar analysis of two smaller cohort of patients^42^,^43^ showed that low GR associates with poor prognosis only in grade 2 and grade 3 patients, but no such association was found in the grade 1 group (Supplementary Fig. 6B), raising the possibility that loss of GR occurs late in breast cancer progression.

To relate these observations to the emerging notion that GR suppresses RTK signalling, we immunostained 362 breast cancer specimens for both GR and the active form of ERK. Tumours were scored, on the one hand, as phospho-ERK positive or negative, and on the other hand as high/medium GR, low GR or undetectable GR levels. This analysis suggested an inverse correlation between GR abundance and ERK activation (Fig. 7c, *P*=0.013). In conclusion, low abundance of GR seems to associate with both higher ERK activation and poorer prognosis.

In summary, we uncovered a complex transcriptional cross-talk between two cardinal signalling pathways, RTKs and GRs. This cross-talk is dominated by the ability of GR to suppress major activators of EGFR signalling and simultaneously activate potent negative feedback loops, which normally restrain RTKs. The observations we made in animal models and analyses of patient specimens favour circadian regulation of RTKs by the HPA neuroendocrine axis. Our model and tumour xenograft studies further offer a strategy to enhance the therapeutic impact of anti-RTK cancer drugs, by means of careful scheduling of drug administration.

## Discussion

In combination, the clades of the 48 NRs and 58 members of the RTK family play pivotal roles in the vast majority of physiological and pathological processes, but their mutual cross-talk remains incompletely understood. This is especially important in the mammary gland, where a complex interplay between RTKs such as EGFR and MET on the one hand and NRs on the other hand controls postnatal morphogenic and functional switches^44^. By analysing EGFR and GR in mammary cells, we concluded that the liganded form of GR employs two mechanistic arms to antagonize EGFR’s signals that normally culminate in cell migration (see a model in Fig. 7d). In line with observations we made in animals, it is predictable that due to circadian oscillations of GR ligands, EGFR signalling is strongly suppressed during the active hours of the day. If extended to other RTKs, the suppressive GR-to-RTK cross-talk might explain the well-characterized ability of GR to inhibit proliferation, angiogenesis and inflammation, processes mediated by partly overlapping sets of RTKs^6^,^45^. In addition, daily oscillations of RTK signalling might explain the recently observed resetting of the circadian clock by a putative pepdidergic factor, which shares with EGFR-specific ligands the ability to control both the serum response factor and actin dynamics^46^. It is also worth noting previously determined significant correlations between serum levels of TGFA and interleukin-6, circadian patterns in wrist activity and serum cortisol, as well as tumour-related symptoms in patients with metastatic colorectal cancer^47^.

GR activation regulates migration of dendritic cells^48^ and antagonizes EGFR during eyelid formation^49^. Likewise, pretreatment of epithelial cells with either cortisol or DEX, but not with mineral steroids, resulted in a downregulation of the EGF-induced expression of 12-lipoxygenase at the mRNA level^50^. Similarly, we report that EGF-induced mammary cell migration is strongly inhibited by DEX. This inhibitory effect might be attributed to antagonizing the ERK/MAPK pathway, because ERK-to-ETS signalling was found to be essential for mammary cell migration^20^. Furthermore, the inhibitory effect of DEX was associated with shorter ERK activation (Fig. 3f), probably due to simultaneous induction of inhibitors of both EGFR and ERK (ERRFI1 and DUSP1, respectively). Thus, the ability of active GRs to inhibit EGF-induced migration is consistent with previous observations and it might engage several complementary mechanisms.

The early EGF-induced onset of positive feedback transcripts (Module B) might be mediated by pre-existing TFs, which undergo rapid covalent modifications following stimulation. These are presumably transrepressed by GR’s tethering, as reported for AP1, STAT5, NF-κB and other TFs. Hence, we analysed the promoters of Module B genes for recurring response elements and identified statistically significant enrichment for binding sites corresponding to IETFs commonly engaged by RTKs (for example, SP1 and ELKs). Thus, in line with early repression capability (Fig. 2c,d), GR seems to selectively engage a subgroup of TFs, including EGR1, GABPA and ELK1, proteins known to regulate mammary cell migration^20^,^51^. Interestingly, some of the validated IETFs transrepressed by GR, such as GABPA and ELK1, belong to the ETS family, while others (for example, SP1 and E2F1) are frequently regulated by growth factors.

Although several cytoplasmic actions of GR have been reported^52^, nuclear activities mediate most physiological actions of GCs. Following translocation to the nucleus GRs enhance or repress transcription of target genes by binding to ‘simple’ response elements, as well as to negative GREs, respectively. Alternatively, GRs might tether themselves to several other TFs^10^. Our results indicate that regulation of EGF-induced transcription by GR employs both modes. Importantly, we discovered that GR signalling harnesses well-characterized network motifs^53^,^54^. These sets of recurring feedback and feed-forward loops serve as building blocks of transcription and signalling networks. In the EGFR network, a group of activation-dependent secreted growth factors, including cytokines and EGFR ligands, both enhance and prolong signalling. Reciprocally, another inducible group, which encodes various cytoplasmic proteins, terminates signalling. To robustly block migration, GR seems to co-opt both regulatory arms of the network: inflammatory cytokines and ligands of EGFR are dampened and concurrently cytoplasmic EGFR inhibitors are enhanced.

Using *Crfr1*-knockout mice^33^, and employing both negative feedback (Module A) (*Errfi1* and *Dusp1*) and positive feedback genes (Module B; *Hbegf* and *Tgfa*) as markers, we obtained *in vivo* evidence supporting circadian regulation of EGFR signalling, similar to the reported diurnal regulation of ERK in the liver^55^. Daily oscillations of RTK signalling are reported here for the first time, and they might relate to a previous report showing that hepatic EGFR’s ligand binding parameters (maximum binding and dissociation constant) undergo rhythmic alterations: in mice, both peaked late in the dark phase and decreased late during the light phase^56^. These oscillations probably have an impact on physiological and pathological processes. For example, some of the most effective asthma medications are inhaled GCs, which inhibit airway inflammation, but also adversely affect epithelium injury repair by suppressing cell migration^57^. Presumably, this dual effect of GCs is due to suppression of specific RTKs involved in tissue repair. Similar interplays seem relevant to psoriasis, fibrosis and atherosclerosis, pathologies influenced by specific RTKs. In particular, an association between cancer progression and inflammation has long been recognized^58^, and accordingly anti-inflammatory drugs can prevent spontaneous tumour formation in people with familial adenomatous polyposis^59^. Importantly, the DEX-inhibited EGFR ligands, such as HB-EGF and TGFA, are well-characterized drivers of autocrine signalling in cancer^25^, whereas the DEX-induced inhibitors of EGFR, such as ERRFI1/MIG6, are collectively downregulated in various tumours^60^. In line with this evidence, data were compiled from breast cancer patients associated high GR with longer survival time, and immunostainings performed on an independent cohort identified an inverse correlation between low GR and activation of the ERK pathway. Notably, our results are consistent with a study that involved complete destruction of the suprachiasmatic nuclei, which ablated the 24-h rest-activity cycle and the daily rhythms of serum corticosterone level. These alterations resulted in a two to three times faster growth of two tumour types^61^.

The emerging diurnal regulation of EGFR activity by GR suggests that tumour progression driven by aberrant forms of RTKs is strongly suppressed during the active phase of the day, but it might reach full impact during the resting phase, when GC levels are low. Our tumour xenograft studies lend support to this model, meaning that cancer patients might derive higher benefit from chronotherapy rather than constant rate infusion of RTK-targeting drugs. Several observations are relevant to this scenario. For example, EGFR and one of its ligands, TGFA, have been implicated in the daily control of locomotor activity^62^. In addition, a flattened diurnal cortisol rhythm can predict breast cancer metastasis^63^, and rodent studies demonstrated that a pan-RTK drug better inhibited tumours if administered in the resting phase rather than in the active phase^64^.

Importantly, circadian physiology, along with gender, clock genes and the cell cycle critically affect outcome of cancer chronotherapeutics^65^, including treatment of colorectal cancer patients with an anti-EGFR antibody^66^. It would be interesting testing the effect of therapy scheduling on the response of tumours to anti-EGFR antibodies such as cetuximab. Although the pharmacokinetics parameters of antibodies are usually longer than those of tyrosine kinase inhibitors, it is notable that a previous study observed improved secondary surgical resectability of colorectal cancer treated with cetuximab and circadian chronomodulated chemotherapy^66^, which is often administered together with DEX. Moreover, careful drug scheduling might improve the reported borderline response of lung cancer to chemotherapy plus cetuximab^67^. Future studies might resolve questions such as the relevance of RTK-targeting chronotherapy to additional monoclonal antibodies and other classes of anticancer drugs.

## Methods

### Cell culture and reagents

MCF10A cells were grown as described^20^ in DME:F12 medium (Gibco BRL, Grand Island, NY) supplemented with 10 μg ml^−1^ insulin, 0.1 μg ml^−1^ cholera toxin, 0.5 μg ml^−1^ HC, 5% heat-inactivated horse serum (Gibco BRL) and 10 ng ml^−1^ EGF. The cells were starved overnight in medium, and thereafter stimulated with EGF (10 ng ml^−1^) or DEX (100 nM). siRNA transfections used Oligofectamine (Invitrogen) and ON-Target SMART (Dharmacon, Lafayette, CO) oligonucleotides. Lapatinib (di-*p*-Toluenesulfonate salt) was purchased from LC Laboratories and formulated as a suspension in 0.5% hydroxypropyl methylcellulose and 0.1% Tween 80. The drug was administered via oral gavage as a single bolus dose. Mig6-specific short hairpin RNA particles were produced in 293FT cells using the corresponding plasmid.

### Lysate preparation and immunoblot analysis

Cell lysates were cleared by centrifugation and resolved by electrophoresis, followed by electrophoretic transfer to a nitrocellulose membrane. Membranes were blocked with TBS-T (Tris-buffered saline containing Tween-20) containing 1% low-fat milk, blotted with a primary antibody for 1 h, washed three times with TBS-T, incubated for 30 min with a secondary antibody linked to horseradish peroxidase and washed with TBS-T. Immunoreactive bands were detected using the ECL reagent (Amersham Pharmacia Biotech, Buckinghamshire, UK).

### RNA isolation, PCR and microarrays

RNA from cultured cells was isolated using the PerfectPure kit and RNA from animal tissues was isolated using the PerfectPure Tissue kit, both from 5 Prime (Hamburg, Germany). Affymetrix GeneChip Human Exon 1.0 ST arrays were used and data were deposited in Gene Expression Omnibus (GSE53405). PCR of pre-mRNA or mRNA used forward primers positioned in the second intron or exon, respectively. All reactions were performed using Power SYBR Green PCR Master Mix (Applied Biosystems, Foster City, CA). Primer sequences are listed in Supplementary Table 1. For high-throughput PCR, we used the Fluidigm BioMark system. The Affymetrix Expression Console was used for analyses of DNA arrays, as described^68^.

### Cell motility assays

Cells (5 × 10^4^ cell per insert) were plated in the upper compartment of a 24-well transwell tray (Corning, Acton, MA) and their migration was assayed^20^. Thereafter, the medium in the lower compartment of the chamber was supplemented with the indicated agents and cells were allowed to migrate for 16 h at 37 °C through the intervening nitrocellulose membrane (8 μm pore size). The filter was later removed and fixed for 15 min in saline containing paraformaldehyde (3%), followed by cell permeabilization in Triton X-100 (0.05% in PBS) and staining with crystal violet. Cells growing on the upper side of the filter were scraped using a cotton swab, and cells located on the bottom side were photographed and counted. For collective migration, 8 × 10^4^ cells were seeded in plastic insets (from Ibidi), and after an overnight incubation the plastic barriers were removed and time-lapse images were recorded. Cell invasion assays were performed using BioCoat Matrigel Invasion Chambers (BD Bioscience, Franklin Lakes, NJ). For tracking, cells were seeded (3 × 10^3^ cells per cm^2^) on collagen-coated micro-slide (from Ibidi). For collective migration, 8 × 10^4^ cells were seeded in plastic insets, and after an overnight incubation the plastic barriers were removed and time-lapse images were recorded under the indicated conditions. The positions of cell nuclei were followed and quantified using ImageJ.

### Analyses of human specimens

Immunohistochemistry of formalin-fixed, paraffin-embedded tissues was performed using the Envision Detection System (DakoCytomation, Carpinteria, CA). Following antigen retrieval, an anti-ERK or an anti-GR antibody (NCL-GCR, Novocastra) was added and incubated overnight at 4 °C. After immunostaining, slides were counterstained with Mayer’s haematoxylin. Two pathologists independently assessed protein levels. Statistical analysis of the data was done using the SPSS suite. Patient survival analysis was performed on a previously described cohort^40^. The *χ*^2^-test was used for association analysis between categorical variables, and a Cox model was fitted to the data using breast cancer-specific death as an endpoint.

### Animal studies

All animal experiments were approved by the Weizmann Institute’s Animal Care and Use Committee. C57BL/6 (Harlan), B6SJL CRFR1 (WT and mutant mice) were maintained under defined flora conditions and at 12-h light–dark cycles. For daily clock studies, female mice (10- to 12-week-old) were divided into two groups; one was maintained in the day–night room, and the second group was located in a special room (with inverted day–light cycles). Mice were let acclimate for at least 1 week before protein and RNA extraction. For tumour xenograft studies, 8-week-old male athymic nude (nu/nu) mice were used and maintained in a specific pathogen-free environment. Animals (*n*=10 per group) were inoculated subcutaneously in the left leg (using a sterile 22-gauge needle) with 5 × 10^6^ N87 cells. Mice were randomized into two groups, daily treated with Lapatinib by oral gavage (40 mg kg^−1^) in the night (2 h after the light off) and in the day (~70 min before light on). Treatments were started 2 weeks after cell injection. Tumour width (*W*) and length (*L*) were measured once a week with a calliper and tumour volume (*V*) was calculated according to the formula: *V*=0.5 × *W*^2^ × *L*.

## Author contributions

M.L. and Y.Y. conceived, designed the experiments and wrote the manuscript. Y.E., A.Z. and M.F. analysed the data. M.L. and S.C. performed the enzyme-linked immunosorbent assays. H.C.-D. performed the directional migration assays. M.K. analysed immunohistochemical results. L.R. and M.S.-S. performed blotting experiments. M.L., G.D. and N.N. assisted the animal studies. K.S. carried out the siRNA screens. M.L. and N.B.-C. performed migration experiments. F.S. supervised immunohistochemical studies. A.C. helped with the CRFR1 mouse model and corticosteroid assays. Y.Y., S.W., R.S. and E.D. supervised the study.

## Additional information

**How to cite this article:** Lauriola, M. *et al*. Diurnal suppression of EGFR signalling by glucocorticoids and implications for tumour progression and treatment. *Nat. Commun.* 5:5073 doi: 10.1038/ncomms6073 (2014).

## Supplementary Material

Supplementary Information Supplementary Figures 1-8 and Supplementary Table 1

## Figures and Tables

**Table 1:** Module B transcription factors predicted to be regulated by GR.

**TF name**	***P-*****value of motif enrichment**	**O/E in ChIP-Seq**	**Median of O/E** ***P-*****value**
GABPA	3.50441E−16	1.845975	3.21E−14
E2F1	4.97513E−14	2.364275	2.53E−26
ELK4	3.53058E−13	2.414661	5.42E−09
MIZF	2.04087E−12		
SP1	1.45558E−10	1.70792	5.81E−09
TFAP2A	1.8067E−09	1.995587	1.51E−10
ELK1	2.55199E−09	1.697793	1.4274E−38
HIF1A::ARNT	2.7473E−08		
Egr1	3.01835E−08	1.649611	5.90E−04
Arnt::Ahr	1.07458E−06		
Klf4	7.93021E−06		
Mycn	1.7274E−05		
Myc	7.72057E−05	2.246657	1.80E−21

ChIP-Seq, chromatin immunoprecipitation-sequencing; EGF, epidermal growth factor; GR, glucocorticoid receptor; O/E, observed relative to expected; TF, transcription factor.

Pscan (http://159.149.160.51/pscan/; Jaspar database) was used to find over-represented TF binding sites in EGF-inducible Module B genes (*n*=593). The Bonferroni corrected *P*-values for multiple testing are shown. In addition, the set of genes was analysed using the Cscan compendium of ChIP-Seq experiments. The respective *P*-values are presented as the median of Bonferroni corrected values.
