# Supplementary material for: Diurnal suppression of EGFR signalling by glucocorticoids and implications for tumour progression and treatment
Source: Nat Commun. 2014 Oct 3;5:5073. doi: 10.1038/ncomms6073 (PMC4205848; doi:10.1038/ncomms6073)
Supplement: Supplementary Information — Supplementary Figures 1-8 and Supplementary Table 1 [file ncomms6073-s1.pdf]

Supplementary Figure 1

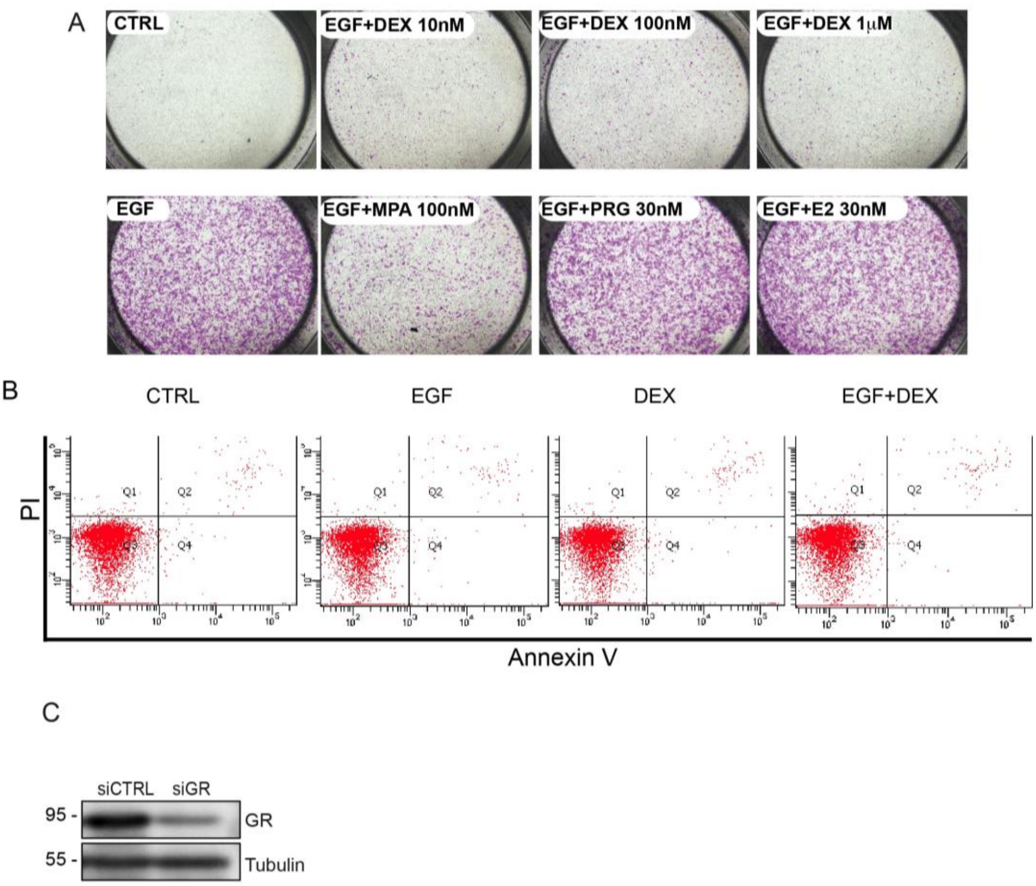

Glucocorticoids inhibit MCF10A cell migration. (**A**) MCF10A cells ($5x10^5$ cells/well) were plated in Transwell chambers and treated with the following agents, either alone or in combinations: EGF (10 ng/ml), DEX (100 nM), estradiol (E2; 30 nM), progesterone (PRG; 30 nM) or medroxyprogesterone acetate (MPA; 100 nM). Shown are representative images of the lower sides of triplicate 8 μm filters, which were stained with crystal violet 20 hours later. The experiment was repeated thrice. (**B**) MCF10A cells pre-treated for 24 hours with of EGF, DEX or the combination. Thereafter, cells were stained for annexin V and the propidium iodide (PI), and later assayed using flow cytometry. (**C**) MCF10A cells were transfected with control siRNA oligonucleotides, or with GR-specific siRNAs, and 48 hours later whole cell extracts were resolved using gel electrophoresis, and immunoblotting with antibodies to GR (ab3580 from Abcam) and tubulin (at approximately 3 μg/ml).

Supplementary Figure 2

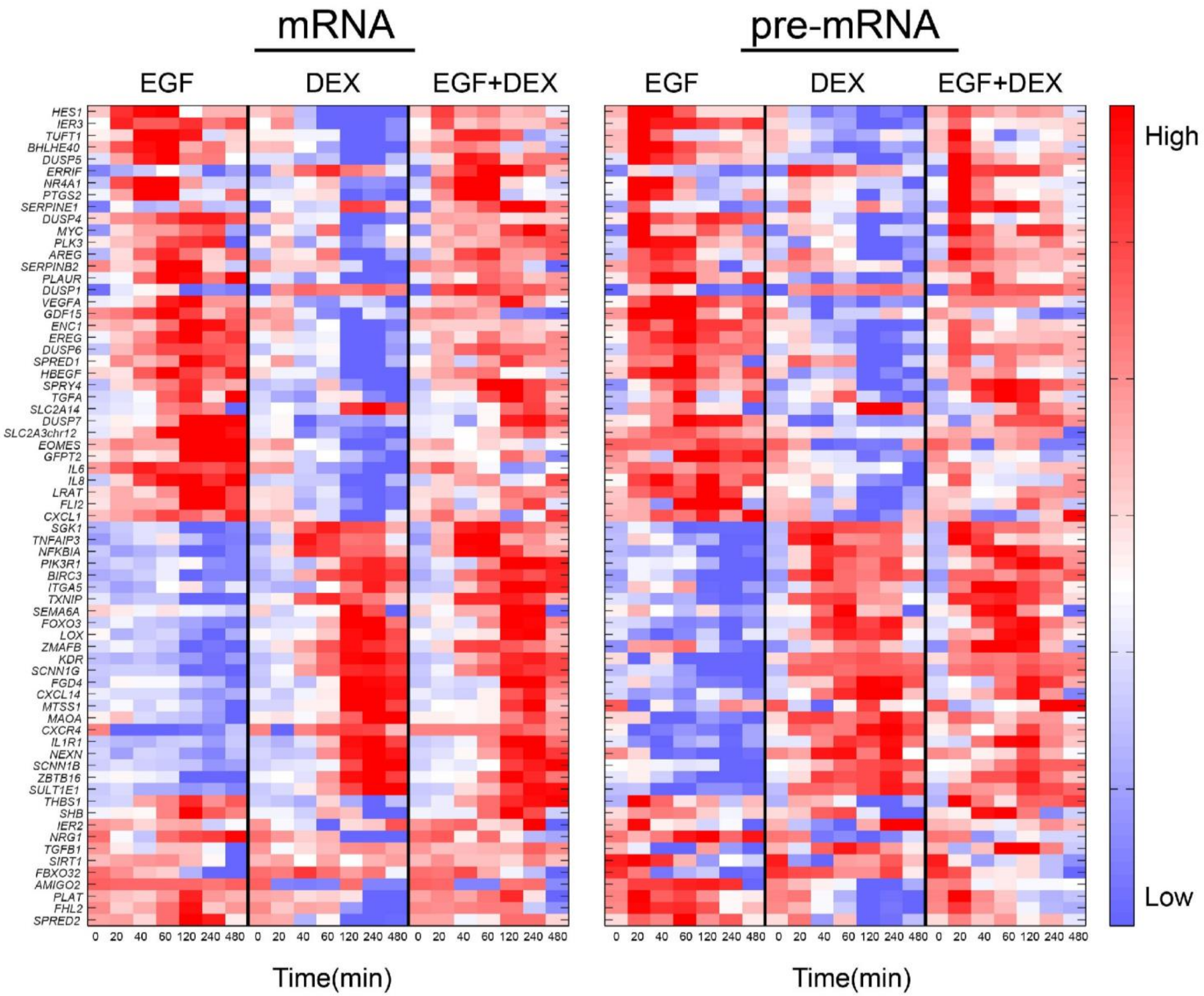

GR signaling regulates EGF-induced transcriptional programs. MCF10A mammary epithelial cells were stimulated with EGF, DEX or the combinations (see Figure 2A) for the indicated time intervals, and RNA samples were processed for high throughput gene expression analyses using real time PCR and microfluidic dynamic arrays (Fluidigm® Real-Time PCR). Both mRNA and pre-mRNA levels were surveyed using specific oligonucleotides. Genes are arranged according to the peak time of the respective mRNA levels.

Supplementary Figure 3

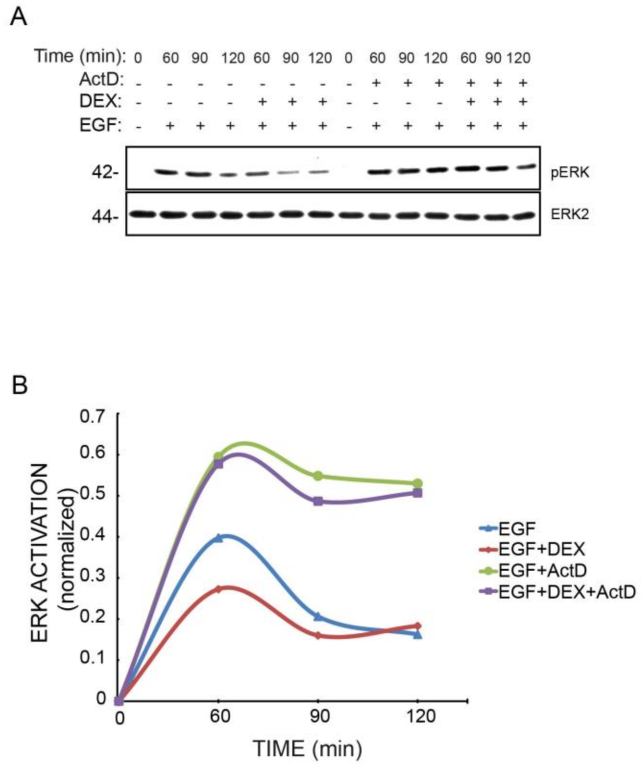

GR signaling enhances de novo synthesis of regulators of the ERK pathway. (**A**) Serum-starved MCF10A cells were pre-incubated for 20 minutes with actinomycin D (1 μg/ml), and thereafter stimulated for the indicated time intervals with EGF or DEX. This was followed by preparation of cell extracts and immunoblotting with a monoclonal antibody the to active (phosphorylated) form of ERK (M8159 from Sigma). The antibody was used at 1:1,000 dilution (approximately 2 μg/ml). (**B**) The pERK signals from A and additional experiments were quantified, normalized to total ERK2 levels and presented.

Supplementary Figure 4

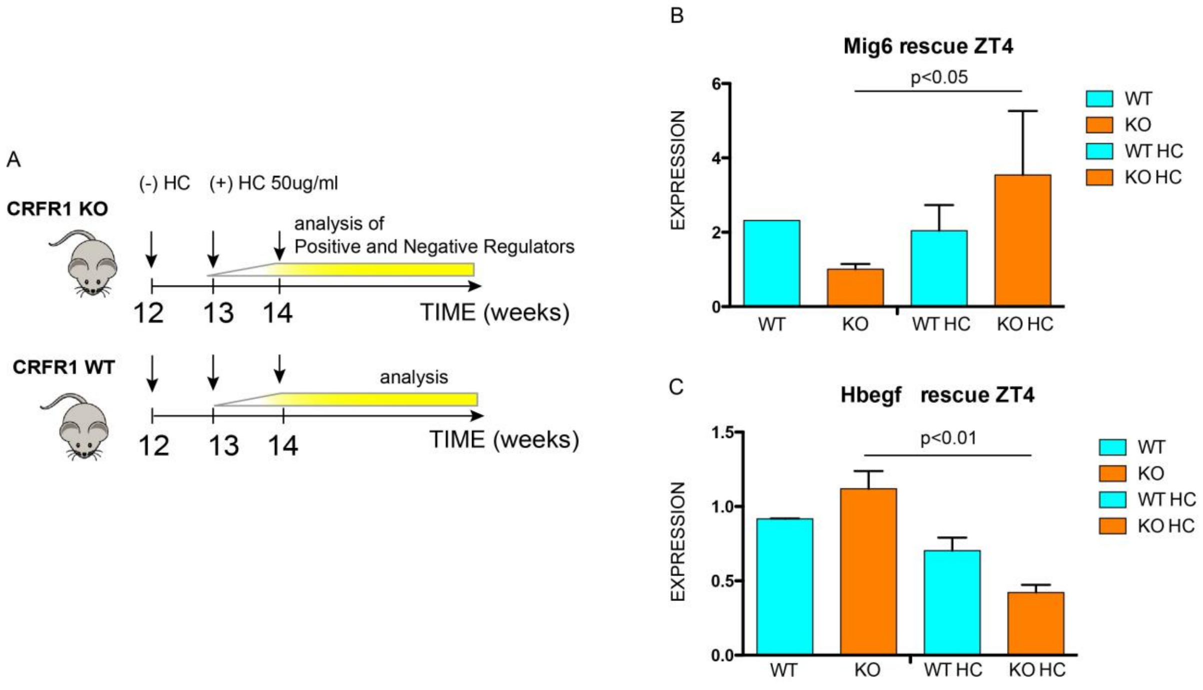

Rescue of the transcriptional phenotype of Crfr1$^{-/-}$ mice using hydrocortisone. (**A**) A scheme of the rescue experiment using Crfr1-depleted animals (KO). (**B**) Wild type animals (WT; n=4) or Crfr1$^{-/-}$ mice (KO; n=4) were treated for a week without or with Hydrocortisone HC (50 µg/ml) in the drinking water. Liver tissues were collected at ZT4 and analyzed using RT-PCR for *Mig6/Errfi1* (a negative regulator) or (**C**) *Hbegf* (a positive regulator). Averages±S.D. values are shown. Pval was calculated by using a 1-way Anova.

Supplementary Figure 5

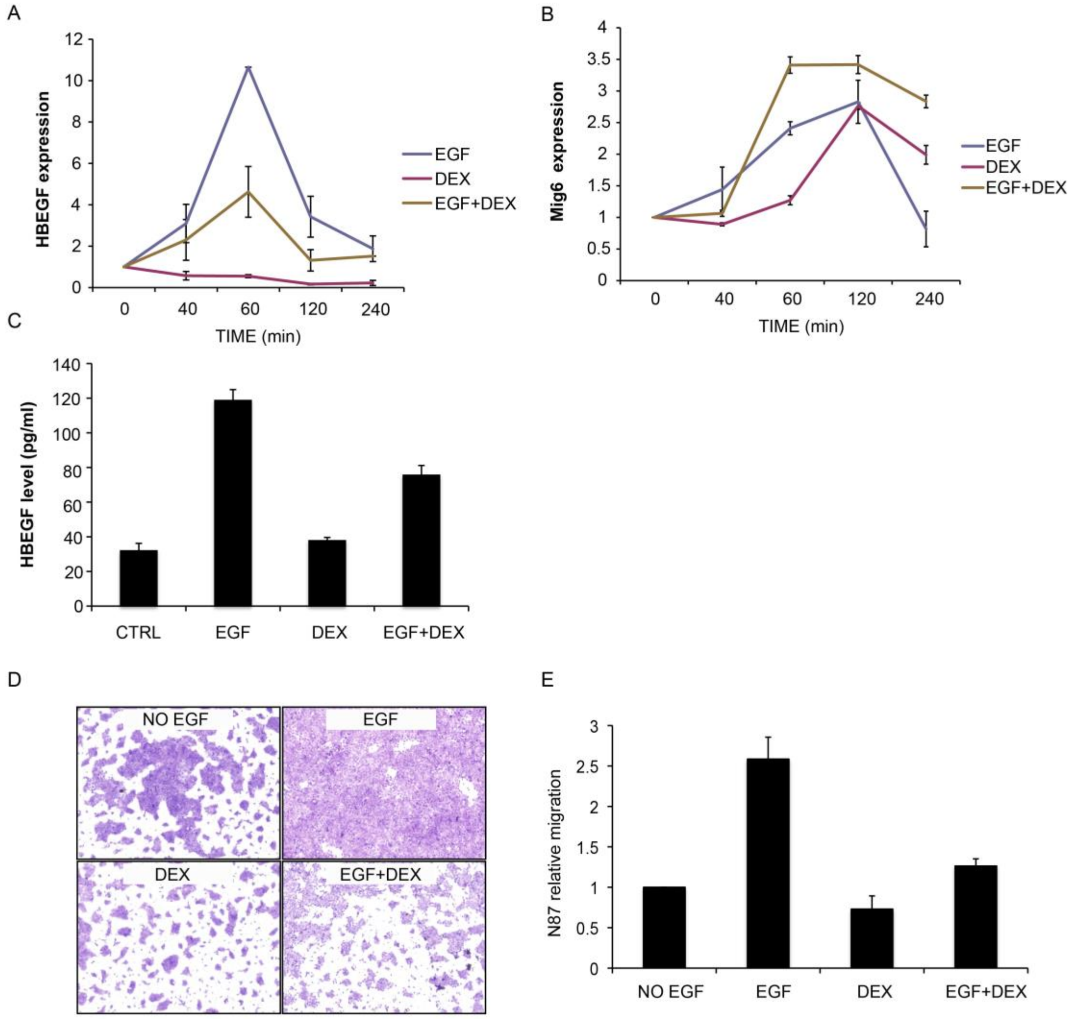

The responses of human N87 gastric cancer cells resemble the responses of mammary cells to EGF and to DEX. (**A** and **B**) Serum-starved N87 cells were treated with EGF or DEX for up to 4 hours, as indicated. qPCR analysis was performed using primers corresponding to either a positive feedback component, HB-EGF (A) or a negative feedback regulator, namely ERRFI1/Mig6 (B). (**C**) N87 cells were serum starved and treated for 4 hours, as indicated. Whole cell extracts were tested for HB-EGF production using ELISA. The results represent biological duplicates performed in technical triplicates. (**D** and **E**) N87 cells growing in transwell plates were treated for 16 hours with EGF (10 ng/ml) or with DEX (100 nM). Crystal violet stained cells that migrated to the lower face of the intervening filter were photographed. The histograms show the results of signal quantification.

Supplementary Figure 6

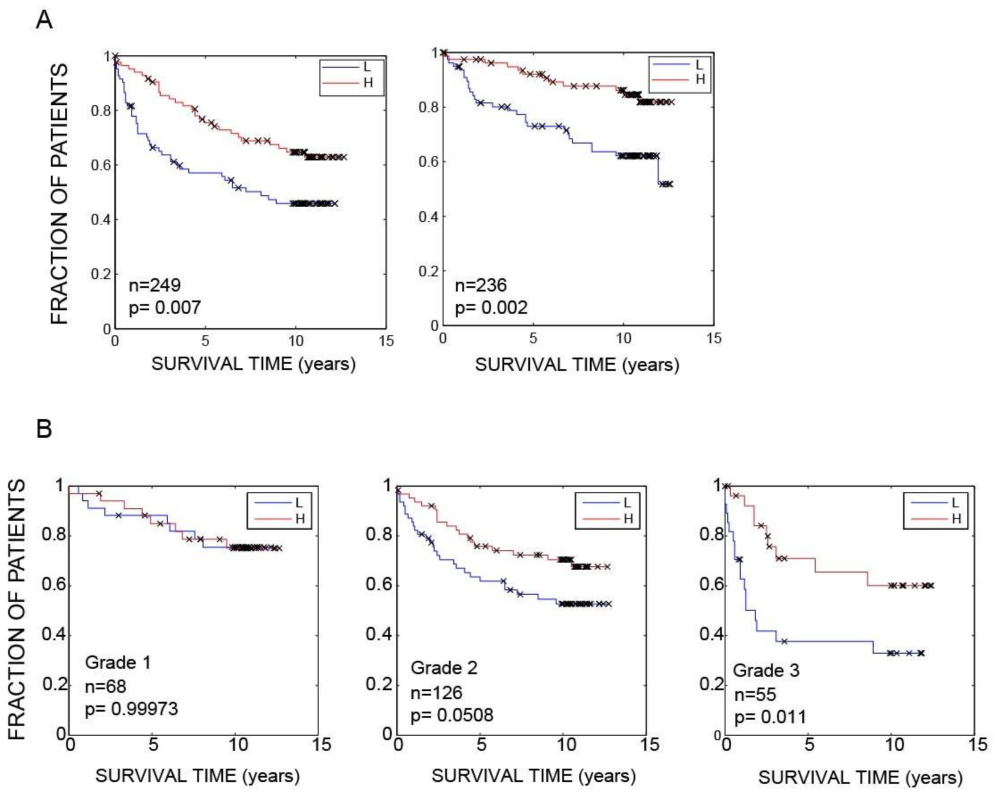

GR abundance predicts survival rate of breast cancer patients and associates with tumor grade. (**A**) Two previously reported breast cancer clinical datasets were analyzed for relapse-free survival (RFS; see main text). Tumors were stratified according to high (red) or low (blue) expression of the NR3C1 (GR) gene. Patient numbers and *p*-values are indicated. (**B**) Patients included in the Ivshina dataset of breast cancer were stratified according to the Elston (NGS) histologic grade, whereby score 1 is the best and 3 is the worst. Note that low GR expression levels associate with shorter survival rates in patients of grades 2 and 3. The expression level of GR was detected in each histological group, and it appears to be lower in grade 2 and 3, relative to grade 1. *p*= 0.0014 (Anova).

Supplementary Figure 7

Western Blots for Figure 3C

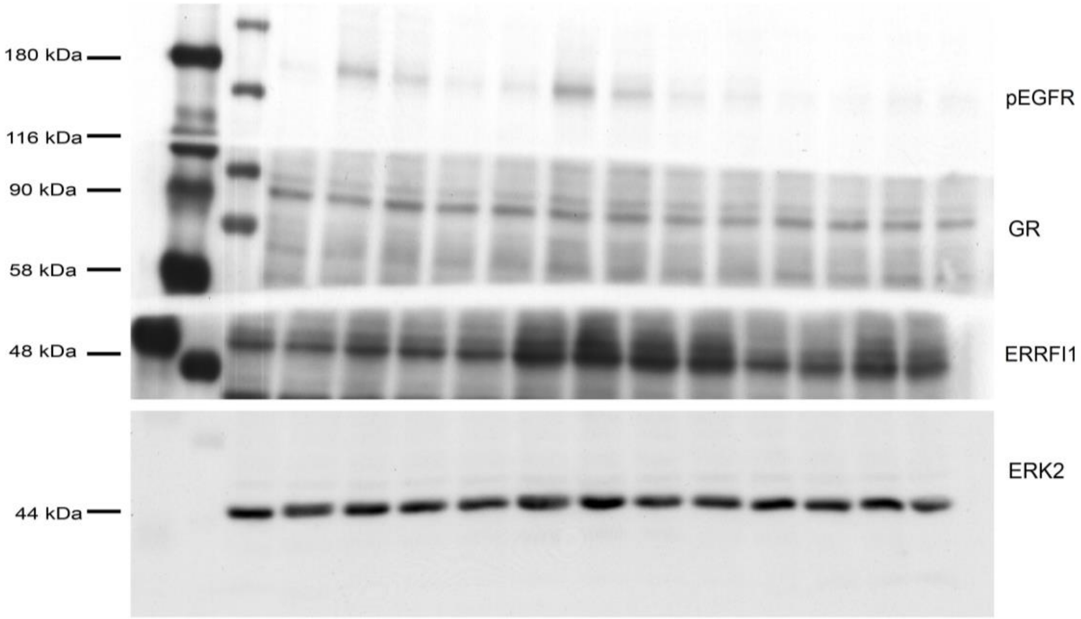

Western Blots for Figure 3E

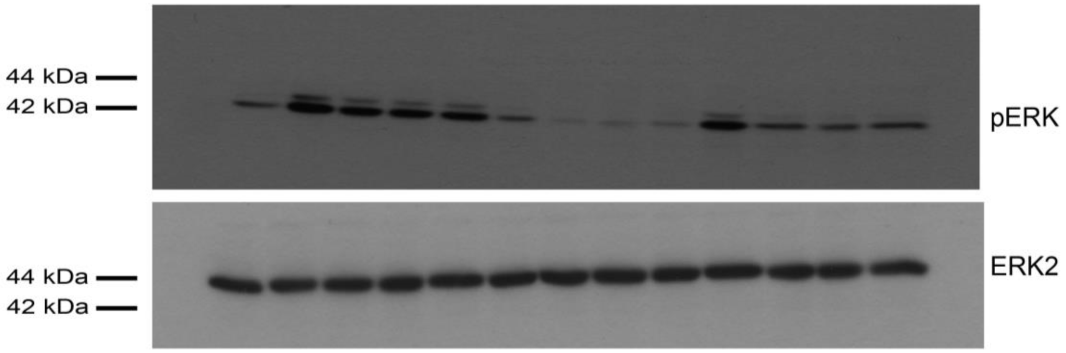

Supplementary Figure 8

Western Blots for Figure 6B

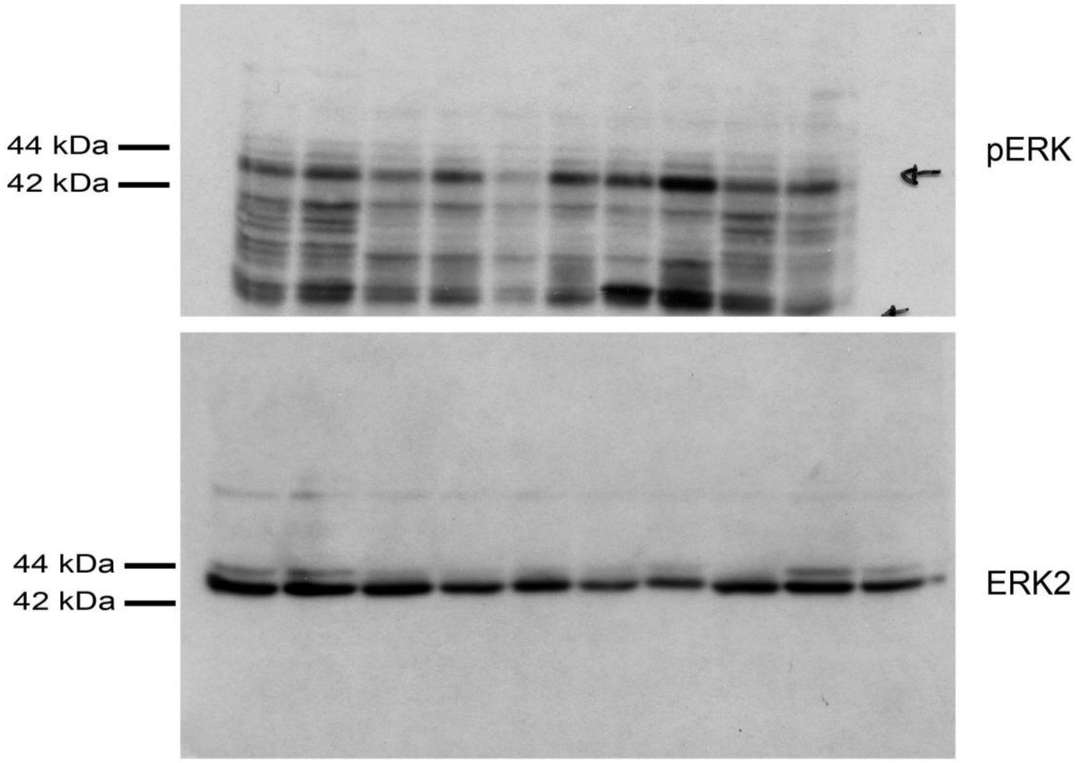

Gene	Sequence 5'---->3'
**AMIGO2_mat**	ATACTGCAGCAGGGCAGAAC
**AMIGO2_pre**	TTTCTGCTTTTTACTCCCTCTGAAT
**AMIGO2_universal**	GAGTCAGATTTCCCCCTCGT
**BHLHE40_mat**	AGACGTGACCGGATTAACGA
**BHLHE40_Pre**	CCCAAAGGTGGGACTTCTCT
**BHLHE40_universal**	CAAGAACCACTGCTTTTTCCA
**CXCL14_mat**	CGCACTGCGAGGAGAAGAT
**CXCL14_pre**	ACCTCATCCTGCTCCGTTTC
**CXCL14_universal**	TTCCAGGCGTTGTACCACTT
**CXCL1_mat**	ATCCTGCATCCCCCATAGTT
**CXCL1_pre**	GAGCAGGGCAGGAGAAGAGT
**CXCL1_universal**	CTTCAGGAACAGCCACCAGT
**DUSP1_mat**	ACTTCATAGACTCCATCAAGAA
**DUSP1_pre**	GAAGGGTGTTTGTCCACTGC
**DUSP1_universal**	CTCGTCCAGCTTGACTCGAT
**DUSP4_mat**	CCACAGAGCCCTTGGACCT
**DUSP4_pre**	CCTGTGCCAAGCACTTTACC
**DUSP4_universal**	GAGGAAGGGAAGGATCTCCA
**ENC1_mat**	TTTGTCAGCACCTGGAAACA
**ENC1_pre**	CATCACACAAATCCTTCATGCT
**ENC1_universal**	AGACTTGGCCTCTCCGAAGT
**EOMES_mat**	CGCCACCAAACTGAGATGAT
**EOMES_pre**	GCCTGTTCTAGGACATCCCAATTA
**EOMES_universal**	TTGTAGTGGGCAGTGGGATT
**EREG_mat**	TCCATCTTCTACAGGCAGTCCT
**EREG_pre**	CTTCCATGAAGGCTGCAGAA
**EREG_universal_R**	AGCCACACGTGGATTGTCTT
**ERRIF1(5' side) P_FW**	TCCTAATGGAGGTATTTCTGAATTGT
**ERRIF1(5' side) P_REV**	CTGGGACATCTCCAAACCTG
**ERRIF1(5' side) mat rev**	CCTCTTCATGTGGTCCCAAG
**ERRIF1(5' side) mat FW**	GCCACTGCTTTGCAGAAAAT
**FGD4_mat**	AGCTGCTCGGAACACTTCAG
**FGD4_pre**	ACCTGATCAGTTTCCCCTATTTCT
**FGD4_universal**	TGGGCACACAGTACAGCAAC
**FLI1_mat**	TCCCTCCTCATGTCATCTCC
**FLI1_pre**	CACGGAAGTGCTGTTGTCAC
**FLI1_Uni**	TCGGTGTGGGAGGTTGTATT
**FOXO3_mat**	CTTCAAGGATAAGGGCGACA
**FOXO3_pre**	CTCGGTTTTGGACCATTCTG
**FOXO3_universal**	TCTTGCCAGTTCCCTCATTC
**GDF15_mat**	GAGCTGGGAAGATTCGAACA
**GDF15_pre**	GTTCCTGGAAAACGGTAGGC
**GDF15_universal**	CGAGAGATACGCAGGTGCAG
**GFPT2_mat**	CCTGTGCCAAGTGTGTGAGA
**GFPT2_pre**	CGGCTGGAGTACAGAGGCTA

**GFPT2_universal**	GACTTCGTGATTATTCCCATCG
**HBEGF_mat**	GCTGTGGTGCTGTCATCTGT
**HBEGF_pre**	CTTTGGAAGGACCTGCTCTG
**HBEGF_universal**	TCATGCCCAACTTCACTTTCT
**IL8_mat**	CGGAAGGAACCATCTCACTG
**IL8_pre**	AAAGGAAGTAGCTGGCAGAGC
**IL8_universal**	AGCACTCCTTGGCAAAACTG
**HES1_mat**	AAGGCGGACATTCTGGAAAT
**HES1_pre**	TGACCCGTCTGTCTCTTTCTG
**HES1_universal**	TACTTCCCCAGCACACTTGG
**IER3_mat_F**	GGACTACGCTCTGGACCTCA
**IER3_mat_R**	AGTGCGGGGAGTCACAGTTA
**IER3_pre_F**	CGACCTGACCTGTCTCCTGT
**IER3_pre_R**	GCAGAAAGAGAAGCCTTTTGG
**IL6_mat**	GCCAGAGCTGTGCAGATGAG
**IL6_pre**	CATCATCCCATAGCCCAGAG
**IL6_universal**	TCAGGGGTGGTTATTGCATC
**IL1R1_mat_fw**	TCATAGCTCTACTGATTTCTTCTCTGG
**IL1R1_mat_rev**	CGAACATCAATTTCATTTGCAG
**IL1R1_pre_fw**	ATTGCTTCCACCCTTCTTCC
**IL1R1_pre_rev**	AGGACAGGGACGAACATCAA
**LOX_mat**	CGCTGTGACATTCGCTACAC
**LOX_pre**	AAAGGTTGACTTTAAATTTGTCTGTTG
**LOX_universal**	CCATTGGGAGTTTTGCTTTG
**MAOA_mat**	TCTGACCAATTTTTCTCTTTTTGC
**MAOA_pre**	GGACAGGGTTGGAGGAAGAA
**MAOA_universal**	TGCCCAGCTCCTTAGACAAG
**NEXN_mat**	CCGAAAGAAGCAAGCTGAAG
**NEXN_pre**	TGGCTAATTCTGTGCCTTTTG
**NEXN_universal**	TGCTGTGTCTTGGTTTTCCTC
**NRG1_mat**	TGGTTCAAGAATGGGAATGAA
**NRG1_pre**	TGACACCACTTTGGTCCTGA
**NRG1_universal**	CTCTCCAGAATCAGCCAGTGA
**PIK3R1_mat**	TGTTGCACCAGGTTCTTCG
**PIK3R1_pre**	GGTGGGATTTTGTTGTTTGC
**PIK3R1_universal**	GGCAAACTGCTCTGCAAGAT
**SCNN1B_mat**	CTCCGTAGGCTTCAAGACCAT
**SCNN1B_pre**	CATTCCTTCCCCCTAACCAG
**SCNN1B_universal**	TCTCCAGGACAGCTTCCATC
**SEMA6A_mat**	AACACTGGCAATGTCAAGCA
**SEMA6A_pre**	TCAACACAGCTAGGGCATGA
**SEMA6A_universal**	TTGTCCTGGCAACGTTTTCT
**SERPINB2_pre**	TTTGATGGCTACTCAGAAGATTCA
**SERPINB2_mat**	TGGGTCAAGACTCAAACCAAA
**SERPINB2_universal**	TGGTATCCCCATCTACAGAACC
**SLC2A14_mat**	CAATGAACTTGTGGCCTGTG

**SLC2A14_pre**	TCAACCAGCTGGGCATAGTT
**SLC2A14_universal**	AGACCCAAGGATGAGTTCCAG
**SPRY4_mat**	GGCGTCTGCGAGTACAGC
**SPRY4_pre**	GGATTAGGCATCCTGCTCAA
**SPRY4_universal**	CTGAGCATCAGGCTGCAAAC
**VEGFA_mat**	AGGAGGAGGGCAGAATCATC
**VEGFA_pre**	GCATTACAGAGCTGGGTGGA
**VEGFA_universal**	AGCTGCGCTGATAGACATCC
**TNFAIP3_mat**	ACCCTGGAAAGCCAGAAGAA
**TNFAIP3_pre**	TGCTGGGTCTTACATGCAGAT
**TNFAIP3_universal**	CTGAACGCCCCACATGTACT
**TBP__F**	CTTCACACGCCAAGAAACAGT
**TBP__R**	GCTGGCCCATAGTGATCTTT
**TGFA_2_pre_F**	CCCTGGAGAGCTAGGGTAACA
**TGFA_2_mat_F**	GTTTTTGGTGCAGGAGGACA
**TGFA_2_universal_R**	CACCAACGTACCCAGAATGG

Supplementary Table 1 Oligonucleotide sequences corresponding to primers used for PCR amplification of the respective pre-mRNA and mature RNA. The universal primer was used with each of the others in PCR reactions.
